# Clinical factors associated with the therapeutic outcome of chemotherapy in very elderly cancer patients

**DOI:** 10.1007/s10147-018-01385-8

**Published:** 2019-01-03

**Authors:** Satoko Ito, Haruyuki Ito, Noriyuki Sato, Yasuo Hirayama, Toshihiko Kusakabe, Takeshi Terui, Kunihiko Ishitani

**Affiliations:** 1 Division of Internal Medicine, Department of Medicine, Higashi-Sapporo Hospital, 3-3-7-35, Higashi Sapporo, Shiroishi-ku, Sapporo, Hokkaido 003-8585 Japan; 2 Tenshi College, 1-30, Kita13 Jo Higashi 3 Chome, 1-30, Higashi-ku, Sapporo, Hokkaido 065-0013 Japan

**Keywords:** Elderly cancer patients, Chemotherapy, RECIST, Comorbidity index, Serum albumin

## Abstract

**Background:**

The purpose of this study was to detect background factors that might be associated with the therapeutic and curative outcome of chemotherapy in elderly cancer patients aged over 75 years.

**Methods:**

A retrospective study was conducted for elderly cancer patients aged over 75 years who had received more than 2 courses of chemotherapy at our hospital. We analyzed the relationships between RECIST outcome and background factors, such as age, sex, clinical TNM stage, pre-treatment history, ECOG performance status, serum albumin, and Charlson comorbidity index using logistic regression analysis.

**Results:**

A total of 103 cancer patients aged over 75 years were analyzed in this study, including 28 with hematological neoplasia, 36 with gastrointestinal tract cancers, 25 with breast cancers, and 14 with other malignancies originating in various tissues. Seventy-one patients (69.1%) had a positive clinical outcome including RECIST CR (complete response), PR (partial response) and SD (stable disease). Multivariate analysis showed that a high serum albumin level of more than 3.5 g/dl and a Charlson comorbidity index score of less than 2 points were positively correlated with a favorable therapeutic outcome.

**Conclusions:**

The results of the current study suggested that serum albumin level and comorbidity index are the principal clinical factors affecting therapeutic outcomes in elderly cancer patients receiving chemotherapy. In the future, these factors may make chemotherapy adaptations, continuity, and effectiveness easier to predict than GA screening.

## Introduction

In Japan, the proportion of the total Japanese population aged over 75 years is estimated to exceed 25% by 2025 [1]. This implies that number of elderly cancer patients will continue increase year by year.

When routinely caring for very elderly cancer patients, it is necessary to pay particular attention to the fact that they form a heterogeneous group with respect to overall health status due to differences in comorbidities, functional status, geriatric syndromes, and socioeconomic aspects resulting in decreased physical reserves [2]. Furthermore, older patients with cancer have usually been under-represented in clinical trials toward the development of new cancer therapies [3]. Thus, clinical and laboratory data for guiding the clinical treatment of these very elderly cancer patients are generally very limited.

Previous reports have suggested that older cancer patients with good clinical performance status are able to tolerate a chemotherapeutic regimen as well as younger patients, particularly when adequate supportive care is provided [4–6]. However, few studies have focused on very elderly cancer patients, particularly those with poor clinical performance status.

Meanwhile, various tools have recently been developed for the geriatric assessment (GA) of cancer patients. However, some of these tools are too time-consuming for routine use, and the practical application of these devices during various clinical treatments is difficult except in large national centers that can engage staff members with various fields of expertise. Furthermore, these tools have not yet been developed to a point at which they can be applied as a means of evaluation even during treatment. In this current study, we attempted to clarify the background factors associated with the therapeutic and curative effects of chemotherapy in elderly cancer patients, particularly those aged over 75 years.

## Methods

### Study design

This was a case–control study. Patients aged over 75 years with various types of malignant tumor receiving chemotherapy treatment at the Higashi-Sapporo Hospital, Sapporo, were extracted randomly from the electronic medical records. Evaluation of clinical outcome was performed using the RECIST criteria and clinical factors that might influence outcome were examined. This study was approved by the Ethical Review Board of Higashi-Sapporo Hospital, Sapporo, Japan.

### Participants

Patients with various types of malignancy, including malignant lymphomas at any clinical TNM stage, diagnosed at the Medical Oncology Unit of Higashi-Sapporo Hospital or affiliated hospitals between June 2013 and July 2016 were enrolled in this study. The eligibility criteria included (1) elderly patients aged over 75 years and (2) receiving more than two courses of standard cancer chemotherapy.

Sociodemographic data such as age and gender were obtained from the electronic medical records. Clinical information including tumor type and clinical TNM stage of cancer, Eastern cooperative oncology group (ECOG) performance status (PS), serum albumin level, specific organ disorders, namely, Charlson comorbidity index (CCI) [7] (Table 1), and previous chemotherapy treatment was collected by a review of medical charts and physician assessment. The following sociodemographic and clinical variables were evaluated: age (continuous variable), gender (male or female), ECOG performance status PS (0–1 or 2–4), clinical TNM stage (stage I–III or IV), tumor type consisting of hematological, mammary, gastrointestinal, and other (lung, oral, soft tissue, renal and gynecologic tumors), serum albumin level, specific organ disorders given as a CCI score, and past chemotherapy history. Some patients were identified with double cancers.

**Table 1:** Charlson comorbidity index [7]

Points	Comorbidity
1	(1) Myocardial infarction
	(2) Congestive heart failure
	(3) Peripheral vascular disease
	(4) Dementia
	(5) Chronic pulmonary disease
	(6) Ulcerative disease
	(7) Mild liver disease
	(8) Diabetes (without complications)
	(9) Cerebrovascular disease
	(10) Collagen disease
2	(1) Diabetes with end-organ damage
	(2) Hemiplegia
	(3) Moderate or severe renal disease
	(4) Second solid tumor (nonmetastatic)
	(5) Leukemia
	(6) Lymphoma, multiple myeloma
3	Moderate or severe liver disease
4	(1) Second metastatic solid tumor
	(2) Acquired immunodeficiency syndrome

### Outcome assessment

Therapeutic effect was determined on the basis of the RECIST criteria and consisted of CR (complete response), PR (partial response), SD (stable disease), and PD (progressive disease). Subject samples were classified into two groups based on the RECIST criteria, with CR, PR, and SD categorized as maintenance or a favorable effect and PD as a negative effect. Treatment effect was also judged in terms of the maintenance of patient quality of life (QOL).

### Statistical analysis

Descriptive statistics were applied for the patient sociodemographic characteristics and clinical information. The sociodemographic and clinical indicators potentially associated with predictor variables were examined separately in the univariate logistic regression model. To confirm statistical significance, multivariate logistic regression analysis was used to determine the relative contribution of the various factors affecting RECIST outcomes. We considered a *p* value less than 0.05 to be statistically significant. Statistical analyses were performed with SPSS 22.0 J for Windows.

## Results

### Patient and clinical characteristics

The clinicopathological characteristics of the 103 patients with malignancies of various origins are summarized in Table 2. The mean age was 80.3 ± 3.9 (range 75–91 years), with 68% of patients female. Fifty-one patients were classified as ECOG PS 0–1 and 52 patients as PS 2-4. Seventy-seven patients were classified as clinical stage IV. With regard to pathohistological type, 36 malignancies were gastrointestinal, 28 were hematological, 25 were mammary, and 14 were neoplasms of other histological origins. Routine laboratory examination of the serum albumin level revealed a mean value of 3.49 ± 0.52 g/dl. To evaluate severe specific organ disorders, CCI at the time of cancer diagnosis was scored, with 29 (28.2%) patients showing a score of > 2 points, indicating a severe specific organ disorder.

**Table 2:** Patient and clinical characteristics

Variable	No. of cases (*n* = 103)
Age, mean ± SD (range of years)	80.3 ± 3.9 (75–91)
Sex (%)	
Male	33 (32.0)
Female	70 (68.0)
ECOG PS, *n* (%)	
0	3 (2.9)
1	48 (46.6)
2	36 (35.0)
3	15 (14.6)
4	1 (1.0)
Stage, *n* (%)	
I	4 (3.9)
II	5 (4.9)
III	17 (16.5)
IV	77 (74.8)
Cancer type, *n* (%)	
Hematological	28 (27.2)
Mammary	25 (24.3)
Gastrointestinal	36 (35.0)
Others^a^	14 (13.6)
Serum albumin (g/dl) mean ± SD	3.49 ± 0.52
Specific organ disorder, *n* (%)^b^	
Yes	29 (28.2)
No	74 (71.8)
Past chemotherapy history, *n* (%)	
Yes	43 (41.7)
No	60 (58.3)

*SD* standard derivation

^a^Includes 9 pulmonary, and one each of oral, sarcoma, renal, gynecological, unknown origin, and double cancer

^b^Charlson comorbidity index (CCI) score of more than 2 points

### Relationship between chemotherapeutic response and clinical parameters

We observed the chemotherapeutic response of each cancer patient as assessed using the RECIST criteria (Table 3). Seventy-one patients (69.1%) of the 103 patients were categorized as CR/PR/SD, while 32 were categorized as PD.

**Table 3:** Summary of chemoherapeutic response as assessed by RECIST criteria

Response	No. of cases (%), *n* = 103
CR	19 (18.4)
PR	29 (28.2)
SD	23 (22.3)
Subtotal	71 (69.1)
PD	32 (30.9)

*CR* complete response, *PR* partial response, *SD* stable disease, *PD* progressive disease

Table 4 shows the findings from the univariate logistic regression analysis of background factors thought to influence the chemotherapeutic effect (RECIST CR/PR/SD) in cancer patients with aged over 75 years. Of the several factors examined, ECOG PS, clinical stage, certain cancer types (such as gastrointestinal cancer), serum albumin level, and specific organ disorder were shown to be significant background factors in the current univariate analysis.

**Table 4:** Univariate logistic regression analysis to identify background factors influencing chemotherapeutic effect in elderly cancer patients

Variable	*β*	Odds ratio (95% CI)	*p* value
Age	− 0.09	0.991 (0.890–1.103)	0.865
Sex, male versus female	− 0.014	0.986 (0.40–2.428)	0.975
ECOG PS, 0–1 versus 2–4	1.230	3.422 (1.383–8.469)	0.008
Stage, I–III versus IV	1.477	4.381 (1.206–15.909)	0.025
Cancer type			
Hematological			0.042
Versus mammary	0.639	1.895 (0.467–7.691)	0.371
Versus gastrointestinal	1.681	5.368 (1.547–18.633)	0.008
Versus others^a^	0.875	2.400 (0.499–11.536)	0.274
Serum albumin, less versus more than 3.5 (g/dl)	− 1.943	0.143 (0.055–0.371)	0.0001
Specific organ disorder (CCI score of more than 2 points), no versus yes	0.927	2.528 (1.023–6.244)	0.044
Past chemotherapy, no versus yes	0.765	2.148 (0.913–5.053)	0.080

*CCI* Charlson comorbidity index

^a^Includes 9 pulmonary, and one each of oral, sarcoma, renal, gynecological, unknown origin, and double cancer

The results of the multivariable logistic regression analysis shown in Table 5 reveal that age, sex, ECOG PS, TNM stage, cancer type, and history of previous treatment were not significant factors affecting antitumor efficacy. However, a serum albumin level of more than 3.5 g/dl (OR = 0.171; 95%CI = 0.055–0.534; *P* = 0.002) and a specific organ disorder CCI score of more than 2 points (OR = 3.365; 95%CI = 1.069–10.596; *P* = 0.038) were significant background factors influencing chemotherapeutic effect. Thus, only serum albumin level and CCI score were found to be positive clinical markers in both the univariate logistic regression and multivariable logistic regression analyses.

**Table 5:** Multivariate logistic regression analysis to identify background factors influencing chemotherapeutic effect in elderly cancer patients

Variable	*β*	Odds ratio (95% CI)	*p* value
Age	− 0.005	0.995 (0.878–1.127)	0.936
Sex, male versus female	0.167	1.182 (0.369–3.790)	0.778
ECOG PS, 0-1 versus 2-4	0.213	1.237 (0.372–4.110)	0.728
Stage, I–III versus IV	0.617	1.854 (0.357–9.634)	0.463
Cancer type			
Hematologyical			0.457
Versus mammary	0.164	1.179 (0.169–8.205)	0.868
Versus gastrointestinal	0.960	2.612 (0.485–14.066)	0.264
Versus others^a^	0.126	1.135 (0.134–9.610)	0.908
Serum albumin, less versus more than 3.5 (g/dl)	− 1.768	0.171 (0.055–0.534)	0.002
Specific organ disorder (CCI score of more than 2 points), no versus yes	1.213	3.365 (1.069–10.596)	0.038
Past chemotherapy, no versus yes	0.668	1.951 (0.666–5.718)	0.223

*CCI* Charlson comorbidity index

^a^Includes 9 pulmonary, and one each of oral, sarcoma, renal, gynecological, unknown origin, and double cancer

## Discussion

In this study, we retrospectively analyzed background factors that could influence the curative effect of chemotherapy for cancer patients aged over 75 years, as assessed by RECIST criteria. As a result, we first suggested that serum albumin level and a CCI score of more than 2 points were correlated with clinical curative effect. Conversely, other factors such as sex, clinical TNM stage, ECOG PS, type of cancer, and past history of previous treatments were found not to be meaningful background factors.

The guidelines of the National Comprehensive Cancer Network, the International Society of Geriatric Oncology, and the European Organization for Research and Treatment of Cancer recommend that all patients with cancer aged over 70 years undergo some form of GA [8–10]. Several reports showed that GA in older cancer patients may help identify health and functional status issues and that several domains of GA are associated with oncological outcomes [11, 12]; for example, cancer-specific geriatric assessment (CSGA) [13], G8 [14] and VES-13 [2]. However, it appears that these assessments rarely cover all elements of the physical and functional status of very elderly patients and/or those with poor performance status.

CRASH [15] and CARG scores [16] have been utilized as assessment tools for side effects and clinical outcome, and it is also thought that overall survival (OS) and progression-free survival (PFS) cannot be used as adequate indicators as very elderly patients have physiologically very limited residual life span. In addition, OS and PFS usually depend on the type and origin of the malignancy as well.

Thus, in this study, we used therapeutic curative effect as an index of clinical outcome. To this end, we utilized laboratory data and common clinical background data to reduce biases arising among responsible staff members. As a result, it was shown that a serum albumin level of more than 3.5 g/dl and a CCI score of less than 2 points afforded objective markers that may be correlated with a favorable clinical outcome.

Serum albumin level is known to be one of the indicators of nutritional status. In cancer patients, it has been reported that a low albumin level is related to serious blood toxicity [17] as well as non-blood toxicity [18] with chemotherapy, and that a low albumin level may be a factor indicative of a poor prognosis [19]. It is also related to mortality in certain cancers [20–23]. Therefore, serum albumin level may have a strong correlation with toxicity, continuation of chemotherapy and clinical prognosis.

Elderly cancer patients have generally an increased prevalence of comorbidities that influence cancer prognosis and treatment tolerance [24–26]. Neugut et al. reported that elderly colon cancer patients with favorable comorbid conditions tended to receive less treatment [27]. There are also some reports, showing that the CCI score is one of the factors influencing prognosis in some types of cancer, including lung cancer [28, 29]. Therefore, it appears that these two factors are reliable prognostic factors with regard to chemotherapeutic outcomes in elderly patients.

Nevertheless, we noticed a few limitations to our current study. First, as the background factors for each patient who received chemotherapy were extremely diverse, such as PS and prognostic factors, and as we respected the wishes of the patients and their families regarding treatment, it is undeniable that the selection criteria for treatment were somewhat ambiguous. Therefore, in the current study, we only analyzed patients who could receive at least two courses of chemotherapy. Second, this retrospective study was carried out in a single institution and several affiliated hospitals, and poor PS patients were given chemotherapy while receiving appropriate palliative care. Patients receiving early palliative care received less aggressive care at the end of life, as compared with patients receiving standard care [30]. Third, we intended to compare the curative effect, as the treatment period varied considerably among cases. Moreover, treatment-free cancer patients were also included in current study, as they met the inclusion criteria for clinical status such as stage IV disease and an ECOG PS of more than 2. Nevertheless, there are many cases that showed a favorable response to chemotherapeutic treatment even among those with stage IV disease and poor PS. Thus, these findings suggest that when serum albumin and comorbidity remain at the levels described above, we can anticipate a favorable clinical chemotherapeutic response even in elderly cancer patients, regardless of clinical stage and ECOG PS. This appeared to be true even among different types of cancers.

The recommendations developed by the expert panel regarding GA-based assessment tools (ASCO Guideline for Geriatric Oncology 2018 [31]) suggested that that clinicians take into account GA results when recommending chemotherapy and that the information be provided to patients and caregivers to guide treatment decision making. Furthermore, clinicians should implement targeted, GA-guided interventions to manage nononcologic problems. Our current results indicate that this tool may be used a simple tool for not only determining adaptations to chemotherapy, but also its possible continuity and prognosis even in medical facilities, where staff members with the necessary expertise for GA assessment are limited or not available.

In conclusion, although further study with a larger number of cases in a multi-institutional setting is required, our present results suggest that patient serum albumin level and comorbidity status have a substantial influence on the chemotherapeutic outcome in very elderly cancer patients, particularly in those aged over 75 years.

## References

[1] Ministry of Health, Labour and Welfare in Japan “Vital Statistics of Japan. Tokyo, Japan: Statistics and Information Department, Minister’s Secretariat, Ministry of Health and Welfare (2012) “Ministry of Internal Affairs and Communications “national census”, National Institute of Population and Social Security Research “Population Projections for Japan” **(in Japanese)**

[2] Decoster L, Van Puyvelde K, Mohile S (2015). Screening tools for multidimensional health problems warranting a geriatric assessment in older cancer patients: an update on SIOG recommendations. Ann Oncol.

[3] Hurria A, Dale W, Mooney M (2014). Designing therapeutic clinical trials for older and frail adults with cancer: U13 conference recommendations. J Clin Oncol.

[4] Christman K, Muss HB, Case LD (1992). Chemotherapy of metastatic breast cancer in the elderly. The Piedmont Oncology Association experience. JAMA.

[5] Sargent DJ, Goldberg RM, Jacobson SD (2001). A pooled analysis of adjuvant chemotherapy for resected colon cancer in elderly patients. N Engl J Med.

[6] Chen H, Cantor A, Meyer J (2003). Can older cancer patients tolerate chemotherapy? A prospective pilot study. Cancer.

[7] Charlson ME, Pompei P, Ales KL (1987). A new method of classifying prognostic comorbidity in longitudinal studies: development and validation. J Chronic Dis.

[8] Carreca I, Balducci L, Extermann M (2005). Cancer in the older person. Cancer Treat Rev.

[9] Extermann M, Aapro M, Bernabei R (2005). Use of comprehensive geriatric assessment in older cancer patients: recommendations from the task force on CGA of the International Society of Geriatric Oncology (SIOG). Crit Rev Oncol Hematol.

[10] Droz JP, Balducci L, Bolla M (2010). Management of prostate cancer in older men: recommendations of a working group of the International Society of Geriatric Oncology. BJU INt.

[11] Puts MT, Hardt J, Monette J (2012). Use of geriatric assessment for older adults in the oncology setting: a systematic review. J Natl Cancer Inst.

[12] Wildiers H, Heeren P, Puts M (2014). International Society of Geriatric Oncology consensus on geriatric assessment in older patients with cancer. J Clin Oncol.

[13] Hurria A, Gupta S, Zauderer M (2005). Developing a cancer-specific geriatric assessment: a feasibility study. Cancer.

[14] Kenis C, Decoster L, Van Puyvelde K (2014). Performance of two geriatric screening tools in older patients with cancer. J Clin Oncol.

[15] Extermann M, Bolder I, Reich RR (2012). Predicting the risk of chemotherapy toxicity in older patients: the chemotherapy risk assessment scale for high-age patients (CRASH) score. Cancer.

[16] Hurria A, Togawa K, Mohile SG (2011). Predicting chemotherapy toxicity in older adults with cancer: a prospective multicenter study. J Clin Oncol.

[17] Alexandre J, Gross-Goupil M, Falissard B (2003). Evaluation of the nutritional and inflammatory status in cancer patients for the risk assessment of severe haematological toxicity following chemotherapy. Ann Oncol.

[18] Murry DJ, Riva L, Poplack DG (1998). Impact of nutrition on pharmacokinetics of anti-neoplastic agents. Int J Cancer Suppl.

[19] Ogino H, Hanibuchi M, Kakiuchi S (2016). Analysis of the prognostic factors of extensive disease small-cell lung cancer patients in Tokushima University Hospital. J Med Invest.

[20] Onate-Ocana LF, Aiello-Crocifoglio V, Gallardo-Rincon D (2007). Serum albumin as a significant prognostic factor for patients with gastric carcinoma. Ann Surg oncol.

[21] Boonpipattanapong T, Chewatanakornkul S (2006). Preoperative carcinoembryonic antigen and albumin in predicting survival in patients with colon and rectal carcinomas. J Clin Gastroenterol.

[22] Siddiqui A, Heinzerling J, Livingston EH (2007). Predictors of early mortality in veteran patients with pancreatic cancer. Am J Surg.

[23] Lis CG, Grutsch JF, Vashi PG (2003). Is serum albumin an independent predictor of survival in patients with breast cancer?. J Parenter Enteral Nutr.

[24] Extermann M (2007). Interaction between comorbidity and cancer. Cancer Control.

[25] Pal SK, Hurria A (2010). Impact of age, sex, and comorbidity on cancer therapy and disease progression. J Clin Oncol.

[26] Sogaard M, Thomsen RW, Bossen KS (2013). The impact of comorbidity on cancer survival: a review. Clin Epidemiol.

[27] Neugut AI, Matasar M, Wang X (2006). Duration of adjuvant chemotherapy for colon cancer and survival among the elderly. J Clin Oncol.

[28] Asmis TR, Ding K, Seymour L (2008). Age and comorbidity as independent prognostic factors in the treatment of non small-cell lung cancer: a review of National Cancer Institute of Canada Clinical Trials Group trials. J Clin Oncol.

[29] Suzuki H, Hanai N, Nishikawa D (2016). The Charlson comorbidity index is a prognostic factor in sinonasal tract squamous cell carcinoma. Jpn J Clin Oncol.

[30] Temel JS, Greer JA, Muzikansky A (2010). Early palliative care for patients with metastatic non-small-cell lung cancer. N Engl J Med.

[31] Supriya G, Mohile W, Dale, Somerfield MR (2018). practical assessment and management of vulnerabilities in older patients receiving chemotherapy: ASCO guideline for geriatric oncology. J Clin Oncol.

